# Supplementary material for: The Canadian VirusSeq Data Portal & Duotang: open resources for SARS-CoV-2 viral sequences and genomic epidemiology
Source: ArXiv. 2024 May 8:arXiv:2405.04734v1. Preprint. [Version 1] (PMC11100916)
Supplement: 1 [file NIHPP2405.04734V1-supplement-1.pdf]

# Supplementary Information: The Canadian VirusSeq Data Portal & Duotang: open resources for SARS-CoV-2 viral sequences and genomic epidemiology

## 1. Supplementary Methods

### 1.1 DNAStack Viral AI network for genomic variant surveillance

Viral AI is the world's first federated network for genomic variant surveillance, developed by DNAstack in response to the COVID-19 pandemic to support discovery and access to SARS-CoV-2 data. DNAstack partnered with the CanCOGeN - VirusSeq project to make the VirusSeq data available through Viral AI and to support lineage assignment for tracking variants.

Viral AI introduces a new way to share and analyze genomics, clinical, administrative, and related data, facilitating insights about transmission, severity, diagnostics and vaccine escape. As an alternative to the centralized model, where data is uploaded to a single vendor-managed database, Viral AI adopts a federated architecture to connect, analyze, and share data without moving it. This model enables faster, more efficient, regulatory compliant, and regionally sovereign data management, enabling viral surveillance efforts to be more equitable, scalable, and sustainable (see figure S1).

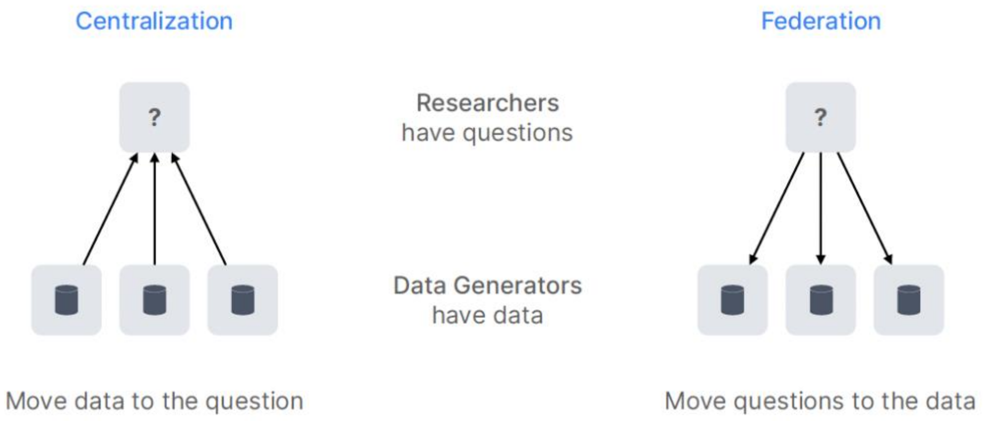


Figure S1: Federation makes it possible to drive discoveries across distributed data without moving it.

Viral AI accelerates science by making data uniformly accessible through a user-friendly graphical interface and powerful programmatic interfaces, integrating data across different sources from around the world alongside VirusSeq, such as NCBI Sequence Read Archive (SRA) and European Center for Disease Prevention and Control, among others. Over one million viral sequences have been added with corresponding assemblies, variant calls, and lineage assignments, all harmonized through an open source bioinformatics pipeline.

Viral AI is powered by a software suite that is compliant with multiple GA4GH standards and facilitates responsible and interoperable genomic and biomedical data sharing including the Data Connect, Data Repository Service, Service Registry, and Service Info standards.

**Publisher** is a data integration and sharing studio that enables data custodians to connect any dataset, from any source, without moving it. Data custodians who contribute data retain administrative control and have transparency into how it's used. DNAstack has connected a number of open source viral genomic data sets using Publisher alongside the VirusSeq data.

**Explorer** is a federated data hub that makes it easier for researchers to find, access, and analyze shared data. With Explorer, researchers can search and perform analyses across a universe of connected datasets through a single user interface. The VirusSeq data is made available in Explorer for researchers to discover, access, and analyze alongside the other connected data sets.

### 1.2 Lineage Assignment Pipeline

An open source bioinformatics pipeline was developed to run lineage assignment on the SARS-CoV-2 genome assemblies obtained from VirusSeq. The resulting lineage assignments, in combination with sample metadata and assemblies, are imported into Viral AI where they are made available over GA4GH standard interfaces.

Assembled SARS-CoV-2 genomes are periodically retrieved from VirusSeq and lineages are assigned using pangolin (Phylogenetic Assignment of Named Global Outbreak LINeages), a tool developed to implement the Pango nomenclature for SARS-CoV-2 lineages. To ensure that lineage assignments are as accurate as possible, the more accurate but slower UShER mode of pangolin is used to assign lineage. Additionally, since pangolin nomenclature and designations are continuously updated as new variants are sequenced and categorized, both pangolin and its underlying databases are updated in sync with new releases. Upon update to pangolin or its databases, all previously assigned lineages are re-assigned using the most up-to-date databases.

In addition to assigning lineage, the pipeline also produces a single-line multifasta and, for each assembly, the set of sites that differs from the SARS-CoV-2 reference genome. The resulting metadata, variant sites, assemblies, and multifasta are processed through an ingestion pipeline and connected to Viral AI where the data is made publicly available for further analysis and interpretation. Following its ingestion into Viral AI, the lineage metadata is retrieved and added to the VirusSeq Data Portal, and remains crucial to the researchers conducting variant surveillance.

## 2. Supplementary Results

### 2.1 List of Selected Contextual Data Fields Available on the Data Portal

- Study ID
- Specimen Collector Sample ID
- Sample Collected By
- Sequence Submitted By
- Submission Date
- Sample Collection Date
- Sample Collection Date Null Reason
- Lineage Name
- Lineage Analysis Software Name
- Lineage Analysis Software Version
- Lineage Analysis Software Data Version
- Scorpio Call
- Scorpio Version

- Geo_loc_name (Country)
- Geo_loc_name (State/province/territory)
- Organism
- Isolate
- Fasta Header Name
- Purpose Of Sampling
- Purpose Of Sampling Details
- Anatomical Material
- Anatomical Part
- Body Product
- Environmental Material
- Environmental Site
- Collection Device
- Collection Method
- Host (Scientific Name)
- Host Disease
- Host Age
- Host Age Null Reason
- Host Age Unit
- Host Age Bin
- Host Gender
- Purpose Of Sequencing
- Purpose Of Sequencing Details
- Sequencing Instrument
- Sequencing Protocol
- Raw Sequence Data Processing Method
- Dehosting Method
- Consensus Sequence Software Name
- Consensus Sequence Software Version
- Breadth Of Coverage Value
- Depth Of Coverage Value
- Reference Genome Accession
- Bioinformatics Protocol
- Gene Name
- Diagnostic Pcr Ct Value
- Diagnostic Pcr Ct Value Null Reason

*For a complete list of Data Portal policies and available contextual data, view* https://virusseq-dataportal.ca/policies

## 3. Consortium and Network Author Information

**Canadian Public Health Laboratory Network (CPHLN) members and staffs having contributed data to the portal**

### Alberta

*Genomes with prefix ABPHL*

Bu J, Croxen M, Deo A, Dieu P, Dong X, Ferrato C, Gavriliuc S, George R, Getachew F, Gill, K, Ie N, Khadka R, Khan F, Koleva P, Lee L, Li V, Lindsay A, Lloyd C, Lynch T, Ma R,

McCullough, E, Mohon A, Murphy S, Obasuyi O, Pabbaraju K, Presbitero A, Rotich S, Shokoples S, Thayer J, Tipples G, Trevor H, Whitehouse M, Wong A, Yu C, Zelyas N

*Genomes in collaboration with University of Calgary (prefix AB-NNNNNN)*

Gordon P, Lam LG, Pabbaraju K, Wong A, Ma R, Li V, Melin A, Tipples G, Berenger B, Zelyas N, Kellner J, Bernier F, Chui L, Croxen M

### British Columbia

*BCCDC Public Health Laboratory*
Natalie Prystajecky, Linda Hoang, John R. Tyson, Dan Fornika, Shannon Russell, Kim MacDonald, Kimia Kamelian, Ana Pacagnella, Corrinne Ng, Loretta Janz, Richard Harrigan, Robert Azana, Tara Newman, Jessica Caleta, Sherrie Wang, Janet Fung, Mel Krajden

### Manitoba

*Cadham Provincial Laboratory collected specimens sequenced at Canada's National Microbiology Lab (NML)***
Paul Van Caeseele, Jared Bullard, David Alexander, Kerry Dust

*Cadham Provincial Laboratory sequenced specimens*
David Alexander, Lori Johnson, Janna Holowick, Joanne Sanders, Adam Hedley, Kerry Dust, Ayo Bolaji, Brooke Cistarelli, Emma Rempel, Paul van Caeseele, Jared Bullard

*Dynacare sequenced specimens*
Hilary Racher, Melissa Desaulnier, Tintu Abraham, Hongbin Li (Impact Genetics, Brampton Ontario)

*NML***
See below for list of NML personnel

### New Brunswick

*Centre Hospitalier Universitaire Georges L. Dumont*
Beauregard AP., Lyons P., Chacko S., Shaw W., Lacroix J., Allain E., Crapoulet N., Garceau R., Desnoyers G.

*NML***
See below for list of NML personnel

### Newfoundland and Labrador

*Newfoundland and Labrador Health Services*
*(previously known as "Newfoundland and Labrador - Eastern Health")*
Robert Needle, Yang Yu, Laura Gilbert, George Zahariadis, Geoffrey Woodland, Chris Corkum, Kerri Smith, Phillip Andrews, Matthew Gilmour

*NML***
See below for list of NML personnel

## Nova Scotia

*QEII Health Sciences Centre**
Todd Hatchette, Jason LeBlanc, Janice Pettipas, Dan Gaston, Greg McCracken
(*data tagged post September 14th should include Allana Loder)

*NML***
See below for list of NML personnel

## Ontario

*Public Health Ontario Laboratory*
Jacob Afelskie,Vanessa G Allen, Rebecca Azzaro, Doonia Bajovic, Philip Banh, Ilse Belgrave , Tom Braukmann, Ashley Carandang, Yao Chen, Claudia Chu, Shawn Clark, Kirby Cronin, Richard de Borja, Rachelle DiTullio, Carla Duncan, Hadi El Roz, Alireza Eshaghi, Nahuel Fittipaldi, Christine Frantz, Dhiraj Gaglani, Nicole Graham, Jonathan B Gubbay, Jennifer L Guthrie, Lawrence Heisler, Daniel Heydari, Mark Horsman, Hadia Hussain, Jason Iraheta, Grace Jeong, Esha Joshi, Sushma Kavikondala, Lisa Kim, Surendra Kumar, Michael Laszloffy, Aimin Li, Michael C.Y. Li, Alex Marchand-Austin, Maria Mariscal, Dean Maxwell, Lisa McTaggart, Fatima Merza, Anupam Mittal, Naadia Mohammed, Esther Nagai, Sandeep Nagra, Shiva Nassori, Paul Nelson, Rima Palencia, John Palmer, Samir N Patel, Stephen Perusini, Nataliya Potapova, Anna Puzinovici, Zarah Rajaei, Christina Rampertab, Himeshi Samarsinghe, Candice Schreiber, Christine Seah, Fatemeh Shaeri, Kapil Shaeri, Kapil Shah, Narisha Shakuralli, Natasha Singh, Karthikeyan Sivaraman, Brenda Stanghini, Ashleigh Sullivan, Vincent Su Bin Cha, Yogi Sundaravadanam, Sarah Teatero, Semra Tibebu, Nobish Varghese, Andre Villegas, Jesse Wang, Matthew Watson, Sichong Xu, Xiao Xu, Kent Young, Sophie Yu, Farhan Yusuf, Sandra Zittermann

*Ontario Institute for Cancer Research*
Jared T. Simpson, Richard de Borja, Paul Krzyzanowski, Bernard Lam, Lawrence Heisler, Michael Laszloffy, Yogi Sundaravadanam, Ilinca Lungu, Lubaina Kothari, Cassandra Bergwerff, Jeremy Johns, Felicia Vincelli, Philip Zuzarte

*McMaster University*
Hooman Derakhshani, Sheridan J.C. Baker, Emily M. Panousis, Ahmed N. Draia, Jalees A. Nasir, Michael G. Surette, Andrew G. McArthur

## Prince Edward Island

*Queen Elizabeth Hospital*
Xiaofeng Ding, Vanessa Arseneau, Kari-Lyn Young
*NML***
See below for list of NML personnel

## Québec

*Laboratoire de Santé Publique du Québec, McGill Génome Sciences Centre; CoVSeq Consortium*
Sandrine Moreira, Jiannis Ragoussis, Guillaume Bourque, Éric Fournier, Aurélie Guilbault, Benjamin Delisle, Dihya Baloul, Inès Levade, Sarah Reiling, Hector Galvez, Paul Stretenowich, Alexandre Montpetit, Michel Roger, Judith Fafard

**Saskatchewan**

*Roy Romanow Provincial Laboratory*
Ryan McDonald, Keith MacKenzie, Kara Loos, Stefani Kary, Meredith Faires, Guruprasad Janga, Rachel DePaulo, Laura Klassen, Alanna Senecal, Amanda Lang, Jessica Minion, Roy Romanow Provincial Laboratory - Molecular Diagnostics

*NML***
See below for list of NML personnel

**Canada's National Microbiology Laboratory (NML)****

Anna Majer, Shari Tyson, Grace Seo, Philip Mabon, Elsie Grudeski, Rhiannon Huzarewich, Russell Mandes, Anneliese Landgraff, Jennifer Tanner, Natalie Knox, Morag Graham, Gary Van Domselaar, Nathalie Bastien, Ruimin Gao, Cody Buchanan, Jasmine Frost, Ameet Bharaj, Cole Slater, Nikki Toledo, Laura Hart, Yan Li, Timothy Booth, Catherine Yoshida, Genevieve Labbe, Adina Bujold, Kara Loos, Jennifer Beirnes, Michael Przybytkowski, Patrick Bastedo, Debra Sorensen, Andrea Tyler, Ana Duggan, Darian Hole, Madison Chapel, Kristen Biggar, Emily Haidl, Chanchal Yadav, Jeff Tuff, Connor Chato, Katherine Eaton, Kirsten Palmier, Molly Pratt, Amber Papineau, Adrian Zetner, Carmen Lìa Murall

Genomics Core Facility at NML
Robotics Support Laboratory at NML

**Academic, Health Network and Research Institutions staffs having contributed data to the Portal**

*Unity Health Toronto*
Ramzi Fattouh, Larissa M. Matukas, Yan Chen, Mark Downing, Trina Otterman, Karel Boissinot, Le Luu

*University Health Network/Mount Sinai Hospital Department of Microbiology*
Marie-Ming Aynaud, Javier Hernandez, Seda Barutcu, Kin Chan, Jessica Bourke, Marc Mazzulli, Tony Mazzulli, Laurence Pelletier, Jeff Wrana, Aimee Paterson, Angel Liu, Allison McGeer

*Kingston Health Sciences Centre and Queen's University*
Prameet M. Sheth, Calvin Sjaarda, Robert Colautti, Katya Douchant

*University of British Columbia*
John R. Tyson, Gabrielle Jayme, Karen Jones, Terrance P. Snutch

*Toronto Invasive Bacterial Diseases Network; Sunnybrook Health Sciences*
Allison McGeer, Patryk Aftanas, Angel Li, Kuganya Nirmalarajah, Emily Panousis, Ahmed Draia, Jalees Nasir, David Richardson, Michael Surette, Samira Mubareka, Andrew G. McArthur

*Eastern Ontario Regional Laboratory Association*
Leanne Mortimer, Hooman Derakhshani, Emily Panousis, Ahmed Draia, Jalees Nasir, Robert Slinger, Andrew G. McArthur

*Western University, CoVizu[vs] Dev Team*
Art Poon, Gopi Gugan, Bonnie Lu, Roux-Cil Ferreira, Molly Liu, Laura Muñoz Baena, Kaitlyn Wade, Navaneeth Mohan, Sandeep Thokala, Abayomi Olabode

**CanCOGeN VirusSeq committees and working groups' members**

*CanCOGeN VirusSeq Implementation Committee*
Terrance Snutch, Fiona Brinkman, Marceline Côté, William Hsiao, Gordon Jolly, Yann Joly, Sharmistha Mishra, Sandrine Moreira, Samira Mubareka, Jared Simpson, Megan Smallwood, Gary Van Domselaar

*CanCOGeN Capacity Building Working Group*
Gary Van Domselaar, Matthew Croxen, Natalie Knox, Celine Nadon, Jennifer Tanner

*CanCOGeN Data Analytics Working Group*
Gary Van Domselaar, Fiona Brinkman, Zohaib Anwar, Robert Beiko, Matieu Bourgey, Guillaume Bourque, Richard de Borja, Ahmed Draia, Jun Duan, Marc Fiume, Dan Fornika, Eric Fournier, Erin Gill, Paul Gordon, Emma Griffiths, Jose Hector Galvez Lopez, Darian Hole, William Hsiao, Jeffrey Joy, Kimia Kamelian, Natalie Knox, Philip Mabon, Finlay Maguire, Tom Matthews, Andrew McArthur, Samir Mechai, Sandrine Moreira, Art Poon, Amos Raphenya, Claire Sevenhuysen, Jared Simpson, Jennifer Tanner, Lauren Tindale, John Tyson, Geoff Winsor, Nolan Woods, Matthew Croxen, Carmen Lìa Murall

*CanCOGeN Ethics and Governance Working Group*
Yann Joly, Fiona Brinkman, Erin Gill, William Hsiao, Hanshi Liu, Sandrine Moreira, Gary Van Domselaar, Ma'n Zawati, Sarah Savić-Kallesøe

*CanCOGeN Metadata Working Group*
William Hsiao, David Alexander, Zohaib Anwar, Nathalie Bastien, Tim Booth, Guillaume Bourque, Fiona Brinkman, Hughes Charest, Caroline Colijn, Matthew Croxen, Guillaume Desnoyers, Rejean Dion, Damion Dooley, Ana Duggan, Leah Dupasquier, Kerry Dust, Eleni Galanis, Emma Garlock, Erin Gill, Gurinder Gopal, Tom Graefenhan, Morag Graham, Emma Griffiths, Linda Hoang, Naveed Janjua, Jeffrey Joy, Kimia Kamelian, Lev Kearney, Natalie Knox, Theodore Kuschak, Jason LeBlanc, Yan Li, Anna Majer, Adel Malek, Ryan McDonald, David Moore, Celine Nadon, Samir Patel, Natalie Prystajecky, Anoosha Sehar, Claire Sevenhuysen, Garrett Sorensen, Laura Steven, Lori Strudwick, Marsha Taylor, Shane Thiessen, Gary Van Domselaar, Adrian Zetner

*CanCOGeN Research Collaborations Working Group*
Fiona Brinkman, Zohaib Anwar, Marceline Côté, Marc Fiume, Laura Gilbert, Erin Gill, Paul Gordon, Yann Joly, Sandrine Moreira, Samira Mubareka, Natalie Prystajecky, Jennifer Tanner, Gary Van Domselaar, Phot Zahariadis,

*CanCOGeN Sequencing Working Group*
Ioannis Ragoussis, Terrance Snutch, Patryk Aftanas, Matthew Croxen, Hooman Derakhshani, Nahuel Fittipaldi, Morag Graham, Andrew McArthur, Sandrine Moreira, Samira Mubareka, Natalie Prystajecky, Ioannis Ragoussis, Jared Simpson, Michael Surette, John Tyson

*CanCOGeN Quality Control Working Group*
Jared Simpson, Mathieu Bourgey, Kodjovi Dodji Mlaga, Nahuel Fittipaldi, Jose Hector Galvez Lopez, Natalie Knox, Genevieve Labbe, Pierre Lyons, Philip Mabon, Finlay Maguire, Anna Majer, Andrew McArthur, Ryan McDonald, Sandrine Moreira, Natalie Prystajecky, Karthikeyan Sivaraman, Kerri Smith, Terrance Snutch, Karthikeyan Sivaraman, Andrea Tyler, John Tyson, Gary Van Domselaar, Matthew Croxen

*Canadian VirusSeq Data Portal (CVDP) Team*
Guillaume Bourque, Lincoln Stein, Christina Yung, Hanshi Liu, Yann Joly, Adrielle Houweling, William Hsiao, Marc Fiume, David Bujold, Erin Gill, Fiona Brinkman, Nithu John, Rosita Bajari, Linda Xiang, Alexandru Lepsa, Jaser Uddin, Justin Richardsson, Leonardo Rivera

Funding for the VirusSeq Data Portal is provided by The Canadian COVID Genomics Network (CanCOGeN), and supported by Genome Canada and Innovation, Science and Economic Development Canada (ISED)
